# Dimethyl 1-(3-cyano­benz­yl)-1*H*-pyrazole-3,5-dicarboxyl­ate

**DOI:** 10.1107/S1600536809014895

**Published:** 2009-04-25

**Authors:** Jie Xiao, Ji-Yuan Yao, Hong Zhao

**Affiliations:** a Ordered Matter Science Research Center, College of Chemistry and Chemical, Engineering, Southeast University, Nanjing 210096, People’s Republic of China

## Abstract

In the mol­ecule of the title compound, C_15_H_13_N_3_O_4_, the dihedral angle between the pyrazole and benzene ring planes is 67.7 (1)°. The crystal structure is stabilized by an intra­molecular C—H⋯O hydrogen bond and two weak inter­molecular C—H⋯O inter­actions.

## Related literature

For the biological activity of pyrazoles, see: Lee *et al.* (1989 ▶); Chambers *et al.* (1985 ▶). For the importance of nitrile derivatives in the synthesis of some heterocyclic mol­ecules, see: Radl *et al.* (2000 ▶). For related structures, see: Fu & Zhao (2007 ▶); Xiao & Zhao (2008*a* ▶,*b* ▶,*c* ▶).
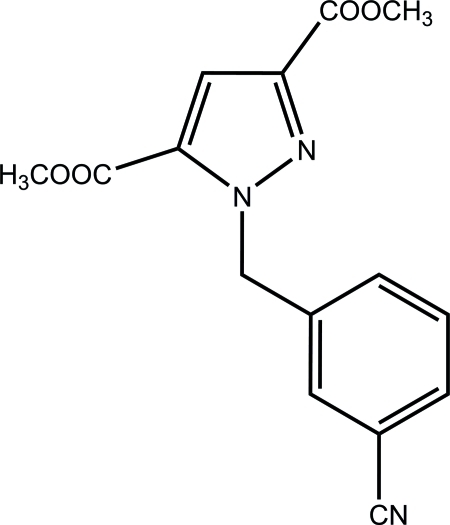


## Experimental

### 

#### Crystal data

C_15_H_13_N_3_O_4_
*M* *_r_* = 299.28
Triclinic, 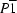

*a* = 8.783 (3) Å
*b* = 9.538 (4) Å
*c* = 9.999 (4) Å
α = 68.42 (3)°
β = 71.79 (4)°
γ = 82.13 (4)°
*V* = 739.7 (5) Å^3^
*Z* = 2
Mo *K*α radiation
μ = 0.10 mm^−1^
*T* = 292 K
0.40 × 0.30 × 0.20 mm

#### Data collection

Rigaku SCXmini diffractometer
Absorption correction: multi-scan (*CrystalClear*; Rigaku, 2005 ▶) *T*_min_ = 0.968, *T*_max_ = 0.979
7555 measured reflections
3338 independent reflections
2093 reflections with *I* > 2σ(*I*)
*R*_int_ = 0.031

#### Refinement

*R*[*F* ^2^ > 2σ(*F* ^2^)] = 0.056
*wR*(*F* ^2^) = 0.147
*S* = 1.05
3338 reflections
201 parameters
H-atom parameters constrained
Δρ_max_ = 0.16 e Å^−3^
Δρ_min_ = −0.16 e Å^−3^

### 

Data collection: *CrystalClear* (Rigaku, 2005 ▶); cell refinement: *CrystalClear*; data reduction: *CrystalClear*; program(s) used to solve structure: *SHELXTL/PC* (Sheldrick, 2008 ▶); program(s) used to refine structure: *SHELXTL/PC*; molecular graphics: *SHELXTL/PC*; software used to prepare material for publication: *SHELXTL/PC*.

## Supplementary Material

Crystal structure: contains datablocks I, global. DOI: 10.1107/S1600536809014895/bx2204sup1.cif

Structure factors: contains datablocks I. DOI: 10.1107/S1600536809014895/bx2204Isup2.hkl

Additional supplementary materials:  crystallographic information; 3D view; checkCIF report

## Figures and Tables

**Table 1:** Hydrogen-bond geometry (Å, °)

*D*—H⋯*A*	*D*—H	H⋯*A*	*D*⋯*A*	*D*—H⋯*A*
C15—H15*B*⋯O1^i^	0.96	2.43	3.363 (3)	163
C9—H9⋯O1^ii^	0.93	2.53	3.348 (3)	147
C6—H6*A*⋯O4	0.97	2.41	2.979 (3)	117

Symmetry codes: (i) 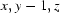; (ii) 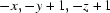.

**Fig. 1.**
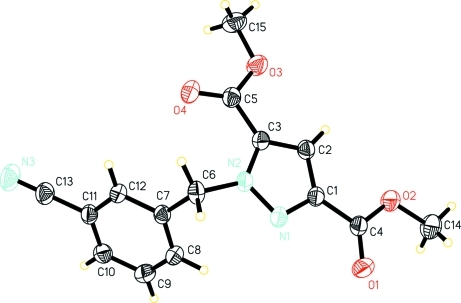
The molecular structure of the title compound, showing the atomic numbering scheme. Displacement ellipsoids are drawn at the 30% probability level.

## supplementary crystallographic information

### Comment

It is well known that many pyrazole-related molecules have received much attention due to their biological activities (Lee *et al.*, 1989; Chambers *et al.*, 1985). In addition, nitrile derivatives are important materials in the synthesis of some heterocyclic molecules (Radl *et al.*, 2000). Recently, we have reported a few benzonitrile compounds (Fu *et al.*, 2007; Xiao *et al.*,2008*a*, 2008*b*,2008*c*). As an extension of our work on the structural characterization of nitrile compounds, the structure of the title compound is reported here. In the molecule of the title compound (Fig. 1) bond lengths and angles have normal values. The dihedral angle between the planes of the pyrazole and phenyl rings is 67.74 (14) °. The molecular conformation is stabilized by an intramolecular C—H···O hydrogen bond and weak intermolecular C—H···O interactions (Table 1)..

### Experimental

Dimethyl 1*H*-Pyrazole-3,5-dicarboxylate (0.185 mg,1 mmol) and 3-(bromomethyl)benzonitrile (0.196 mg,1 mmol) were dissolved in acetone in the presence of K_2_CO_3_ (0.138 mg,1 mmol) and heated under reflux for 1 d. After the mixture was cooled to room temperature, the solution was filtered and the solvent removed in vacuum to afford a white precipitate of the title compound. Colourless crystals suitable for X-ray diffraction were obtained from a solution of 100 mg in 15 ml diethylether by slow evaporation after 7 d.

### Refinement

All H atoms were detected in a difference Fourier map but were placed in calculated positions and refined using a riding motion approximation, with C_aryl_—H, C_methylene_—H, C_methyl_—H = 0.93, 097 and 0.96 Å. *U*_iso_(H_aryl_)=1.2*U*_eq_(C_aryl_); *U*_iso_(H_methylene_)=1.2*U*_eq_(C_methylene_); *U*_iso_(H_methyl_)=1.5*U*_eq_(C_methyl_).

### Crystal data

**Table:**

C_15_H_13_N_3_O_4_	*Z* = 2
*M**_r_* = 299.28	*F*(000) = 312
Triclinic, *P*1	*D*_x_ = 1.344 Mg m^−^^3^
Hall symbol: -P 1	Mo *K*α radiation, λ = 0.71073 Å
*a* = 8.783 (3) Å	Cell parameters from 1442 reflections
*b* = 9.538 (4) Å	θ = 2.4–27.3°
*c* = 9.999 (4) Å	µ = 0.10 mm^−^^1^
α = 68.42 (3)°	*T* = 292 K
β = 71.79 (4)°	Block, colorless
γ = 82.13 (4)°	0.40 × 0.30 × 0.20 mm
*V* = 739.7 (5) Å^3^	

### Data collection

**Table:**

Rigaku SCXmini diffractometer	3338 independent reflections
Radiation source: fine-focus sealed tube	2093 reflections with *I* > 2σ(*I*)
graphite	*R*_int_ = 0.031
Detector resolution: 13.6612 pixels mm^-1^	θ_max_ = 27.4°, θ_min_ = 2.3°
ω scans	*h* = −11→11
Absorption correction: multi-scan (*CrystalClear*; Rigaku, 2005)	*k* = −12→12
*T*_min_ = 0.968, *T*_max_ = 0.979	*l* = −12→12
7555 measured reflections	

### Refinement

**Table:**

Refinement on *F*^2^	Primary atom site location: structure-invariant direct methods
Least-squares matrix: full	Secondary atom site location: difference Fourier map
*R*[*F*^2^ > 2σ(*F*^2^)] = 0.056	Hydrogen site location: inferred from neighbouring sites
*wR*(*F*^2^) = 0.147	H-atom parameters constrained
*S* = 1.05	*w* = 1/[σ^2^(*F*_o_^2^) + (0.0636*P*)^2^ + 0.0696*P*] where *P* = (*F*_o_^2^ + 2*F*_c_^2^)/3
3338 reflections	(Δ/σ)_max_ < 0.001
201 parameters	Δρ_max_ = 0.16 e Å^−^^3^
0 restraints	Δρ_min_ = −0.16 e Å^−^^3^

### Special details

**Table:**

Geometry. All e.s.d.'s (except the e.s.d. in the dihedral angle between two l.s. planes) are estimated using the full covariance matrix. The cell e.s.d.'s are taken into account individually in the estimation of e.s.d.'s in distances, angles and torsion angles; correlations between e.s.d.'s in cell parameters are only used when they are defined by crystal symmetry. An approximate (isotropic) treatment of cell e.s.d.'s is used for estimating e.s.d.'s involving l.s. planes.
Refinement. Refinement of *F*^2^ against ALL reflections. The weighted *R*-factor *wR* and goodness of fit *S* are based on *F*^2^, conventional *R*-factors *R* are based on *F*, with *F* set to zero for negative *F*^2^. The threshold expression of *F*^2^ > σ(*F*^2^) is used only for calculating *R*-factors(gt) *etc*. and is not relevant to the choice of reflections for refinement. *R*-factors based on *F*^2^ are statistically about twice as large as those based on *F*, and *R*- factors based on ALL data will be even larger.

### Fractional atomic coordinates and isotropic or equivalent isotropic displacement parameters (Å^2^)

**Table:**

	*x*	*y*	*z*	*U*_iso_*/*U*_eq_	
C1	0.2466 (2)	0.1608 (2)	0.4437 (2)	0.0473 (5)	
C2	0.2324 (2)	0.0151 (2)	0.4487 (2)	0.0485 (5)	
H2	0.2544	−0.0753	0.5185	0.058*	
C3	0.1787 (2)	0.0343 (2)	0.3276 (2)	0.0453 (5)	
C4	0.2972 (2)	0.2120 (3)	0.5461 (2)	0.0530 (5)	
C5	0.1399 (2)	−0.0794 (2)	0.2763 (2)	0.0516 (5)	
C6	0.1126 (2)	0.2702 (2)	0.1210 (2)	0.0513 (5)	
H6A	0.1388	0.2105	0.0561	0.062*	
H6B	0.1713	0.3630	0.0669	0.062*	
C7	−0.0655 (2)	0.3076 (2)	0.1568 (2)	0.0441 (5)	
C8	−0.1362 (3)	0.3905 (2)	0.2511 (2)	0.0548 (5)	
H8	−0.0735	0.4208	0.2948	0.066*	
C9	−0.2976 (3)	0.4282 (3)	0.2806 (3)	0.0618 (6)	
H9	−0.3429	0.4818	0.3453	0.074*	
C10	−0.3928 (3)	0.3868 (2)	0.2143 (2)	0.0564 (6)	
H10	−0.5012	0.4135	0.2327	0.068*	
C11	−0.3234 (2)	0.3050 (2)	0.1206 (2)	0.0501 (5)	
C12	−0.1607 (2)	0.2639 (2)	0.0923 (2)	0.0476 (5)	
H12	−0.1163	0.2074	0.0303	0.057*	
C13	−0.4218 (3)	0.2621 (3)	0.0498 (3)	0.0625 (6)	
C14	0.3840 (4)	0.1361 (3)	0.7627 (3)	0.0947 (10)	
H14A	0.4685	0.2077	0.7128	0.142*	
H14B	0.4199	0.0470	0.8309	0.142*	
H14C	0.2922	0.1797	0.8174	0.142*	
C15	0.1386 (3)	−0.3432 (3)	0.3310 (3)	0.0756 (7)	
H15A	0.0277	−0.3388	0.3343	0.113*	
H15B	0.1612	−0.4358	0.4042	0.113*	
H15C	0.2046	−0.3388	0.2329	0.113*	
N1	0.2055 (2)	0.26560 (19)	0.32636 (19)	0.0517 (4)	
N2	0.16398 (19)	0.18644 (18)	0.25641 (17)	0.0460 (4)	
N3	−0.4990 (3)	0.2293 (3)	−0.0067 (3)	0.0878 (7)	
O1	0.2979 (2)	0.34090 (19)	0.5354 (2)	0.0816 (6)	
O2	0.3407 (2)	0.09684 (17)	0.65206 (18)	0.0724 (5)	
O3	0.1723 (2)	−0.21721 (16)	0.36374 (17)	0.0659 (5)	
O4	0.0863 (2)	−0.05489 (18)	0.1728 (2)	0.0769 (5)	

### Atomic displacement parameters (Å^2^)

**Table:**

	*U*^11^	*U*^22^	*U*^33^	*U*^12^	*U*^13^	*U*^23^
C1	0.0473 (12)	0.0513 (12)	0.0480 (12)	−0.0011 (9)	−0.0202 (9)	−0.0170 (9)
C2	0.0510 (12)	0.0492 (12)	0.0461 (12)	−0.0012 (9)	−0.0194 (9)	−0.0126 (9)
C3	0.0429 (11)	0.0480 (11)	0.0461 (11)	−0.0024 (8)	−0.0145 (9)	−0.0153 (9)
C4	0.0528 (13)	0.0599 (14)	0.0562 (13)	0.0044 (10)	−0.0243 (10)	−0.0258 (11)
C5	0.0471 (12)	0.0576 (13)	0.0532 (13)	−0.0022 (9)	−0.0164 (10)	−0.0206 (10)
C6	0.0494 (12)	0.0601 (13)	0.0435 (12)	−0.0053 (9)	−0.0207 (9)	−0.0094 (9)
C7	0.0505 (12)	0.0415 (11)	0.0400 (11)	−0.0054 (8)	−0.0182 (9)	−0.0078 (8)
C8	0.0627 (14)	0.0541 (13)	0.0594 (13)	−0.0035 (10)	−0.0279 (11)	−0.0232 (10)
C9	0.0677 (15)	0.0597 (14)	0.0658 (15)	0.0024 (11)	−0.0194 (12)	−0.0314 (12)
C10	0.0512 (13)	0.0570 (13)	0.0634 (14)	0.0023 (10)	−0.0194 (11)	−0.0219 (11)
C11	0.0499 (12)	0.0522 (12)	0.0526 (12)	−0.0029 (9)	−0.0233 (10)	−0.0151 (9)
C12	0.0529 (12)	0.0511 (12)	0.0459 (11)	−0.0005 (9)	−0.0206 (9)	−0.0195 (9)
C13	0.0509 (13)	0.0758 (16)	0.0698 (16)	0.0021 (11)	−0.0254 (12)	−0.0295 (12)
C14	0.140 (3)	0.098 (2)	0.0809 (19)	0.0188 (18)	−0.075 (2)	−0.0427 (16)
C15	0.097 (2)	0.0552 (15)	0.0804 (18)	−0.0088 (13)	−0.0245 (15)	−0.0280 (13)
N1	0.0544 (10)	0.0532 (10)	0.0552 (11)	−0.0020 (8)	−0.0259 (8)	−0.0185 (8)
N2	0.0464 (9)	0.0503 (10)	0.0446 (9)	−0.0034 (7)	−0.0205 (7)	−0.0131 (7)
N3	0.0656 (14)	0.124 (2)	0.1012 (18)	0.0028 (13)	−0.0437 (13)	−0.0557 (16)
O1	0.1237 (16)	0.0589 (11)	0.0919 (13)	0.0097 (10)	−0.0644 (12)	−0.0358 (9)
O2	0.1047 (14)	0.0650 (10)	0.0687 (11)	0.0110 (9)	−0.0542 (10)	−0.0268 (8)
O3	0.0894 (12)	0.0511 (9)	0.0636 (10)	−0.0076 (8)	−0.0321 (9)	−0.0164 (7)
O4	0.1025 (14)	0.0705 (11)	0.0837 (12)	0.0033 (9)	−0.0579 (11)	−0.0317 (9)

### Geometric parameters (Å, °)

**Table:**

C1—N1	1.345 (2)	C8—H8	0.9300
C1—C2	1.393 (3)	C9—C10	1.387 (3)
C1—C4	1.482 (3)	C9—H9	0.9300
C2—C3	1.375 (2)	C10—C11	1.381 (3)
C2—H2	0.9300	C10—H10	0.9300
C3—N2	1.369 (2)	C11—C12	1.397 (3)
C3—C5	1.475 (3)	C11—C13	1.452 (3)
C4—O1	1.195 (2)	C12—H12	0.9300
C4—O2	1.325 (2)	C13—N3	1.143 (3)
C5—O4	1.201 (2)	C14—O2	1.455 (3)
C5—O3	1.334 (3)	C14—H14A	0.9600
C6—N2	1.471 (2)	C14—H14B	0.9600
C6—C7	1.514 (3)	C14—H14C	0.9600
C6—H6A	0.9700	C15—O3	1.444 (3)
C6—H6B	0.9700	C15—H15A	0.9600
C7—C12	1.385 (2)	C15—H15B	0.9600
C7—C8	1.395 (3)	C15—H15C	0.9600
C8—C9	1.380 (3)	N1—N2	1.347 (2)
			
N1—C1—C2	111.68 (17)	C10—C9—H9	119.8
N1—C1—C4	118.44 (18)	C11—C10—C9	118.7 (2)
C2—C1—C4	129.88 (18)	C11—C10—H10	120.6
C3—C2—C1	104.90 (17)	C9—C10—H10	120.6
C3—C2—H2	127.6	C10—C11—C12	121.23 (18)
C1—C2—H2	127.6	C10—C11—C13	119.1 (2)
N2—C3—C2	107.05 (17)	C12—C11—C13	119.63 (19)
N2—C3—C5	123.21 (17)	C7—C12—C11	119.83 (19)
C2—C3—C5	129.74 (18)	C7—C12—H12	120.1
O1—C4—O2	124.09 (19)	C11—C12—H12	120.1
O1—C4—C1	124.3 (2)	N3—C13—C11	179.5 (3)
O2—C4—C1	111.57 (19)	O2—C14—H14A	109.5
O4—C5—O3	124.0 (2)	O2—C14—H14B	109.5
O4—C5—C3	126.4 (2)	H14A—C14—H14B	109.5
O3—C5—C3	109.59 (18)	O2—C14—H14C	109.5
N2—C6—C7	112.47 (16)	H14A—C14—H14C	109.5
N2—C6—H6A	109.1	H14B—C14—H14C	109.5
C7—C6—H6A	109.1	O3—C15—H15A	109.5
N2—C6—H6B	109.1	O3—C15—H15B	109.5
C7—C6—H6B	109.1	H15A—C15—H15B	109.5
H6A—C6—H6B	107.8	O3—C15—H15C	109.5
C12—C7—C8	118.64 (19)	H15A—C15—H15C	109.5
C12—C7—C6	120.28 (18)	H15B—C15—H15C	109.5
C8—C7—C6	121.05 (17)	C1—N1—N2	104.90 (16)
C9—C8—C7	121.12 (19)	N1—N2—C3	111.47 (15)
C9—C8—H8	119.4	N1—N2—C6	118.28 (16)
C7—C8—H8	119.4	C3—N2—C6	130.24 (16)
C8—C9—C10	120.4 (2)	C4—O2—C14	115.33 (19)
C8—C9—H9	119.8	C5—O3—C15	117.10 (18)
			
N1—C1—C2—C3	−0.4 (2)	C9—C10—C11—C13	−179.5 (2)
C4—C1—C2—C3	179.1 (2)	C8—C7—C12—C11	0.9 (3)
C1—C2—C3—N2	0.3 (2)	C6—C7—C12—C11	−177.04 (18)
C1—C2—C3—C5	−179.23 (19)	C10—C11—C12—C7	−1.1 (3)
N1—C1—C4—O1	3.2 (3)	C13—C11—C12—C7	178.51 (18)
C2—C1—C4—O1	−176.3 (2)	C2—C1—N1—N2	0.3 (2)
N1—C1—C4—O2	−177.21 (18)	C4—C1—N1—N2	−179.24 (16)
C2—C1—C4—O2	3.3 (3)	C1—N1—N2—C3	−0.1 (2)
N2—C3—C5—O4	−3.1 (3)	C1—N1—N2—C6	−179.84 (16)
C2—C3—C5—O4	176.4 (2)	C2—C3—N2—N1	−0.1 (2)
N2—C3—C5—O3	177.39 (18)	C5—C3—N2—N1	179.45 (17)
C2—C3—C5—O3	−3.1 (3)	C2—C3—N2—C6	179.55 (18)
N2—C6—C7—C12	−124.57 (19)	C5—C3—N2—C6	−0.9 (3)
N2—C6—C7—C8	57.5 (2)	C7—C6—N2—N1	−87.7 (2)
C12—C7—C8—C9	0.2 (3)	C7—C6—N2—C3	92.6 (2)
C6—C7—C8—C9	178.21 (19)	O1—C4—O2—C14	2.8 (4)
C7—C8—C9—C10	−1.3 (3)	C1—C4—O2—C14	−176.8 (2)
C8—C9—C10—C11	1.1 (3)	O4—C5—O3—C15	−0.6 (3)
C9—C10—C11—C12	0.1 (3)	C3—C5—O3—C15	178.93 (18)

### Hydrogen-bond geometry (Å, °)

**Table:**

*D*—H···*A*	*D*—H	H···*A*	*D*···*A*	*D*—H···*A*
C15—H15B···O1^i^	0.96	2.43	3.363 (3)	163
C9—H9···O1^ii^	0.93	2.53	3.348 (3)	147
C6—H6A···O4	0.97	2.41	2.979 (3)	117

Symmetry codes: (i) *x*, *y*−1, *z*; (ii) −*x*, −*y*+1, −*z*+1.
